# Review on the Role of Host Immune Response in Protection and Immunopathogenesis during Cutaneous Leishmaniasis Infection

**DOI:** 10.1155/2020/2496713

**Published:** 2020-06-18

**Authors:** Teshager Dubie, Yasin Mohammed

**Affiliations:** ^1^College of Veterinary Medicine, Samara University, P.O. Box 132, Samara, Ethiopia; ^2^College of Animal Science, Mekdela Amba University, P.O. Box 1362, Mekdela, Ethiopia

## Abstract

Cutaneous leishmaniasis (CL) is a major public health problem worldwide and spreads to human via the bite of sand flies during blood meal. Following its inoculation, the promastigotes are immediately taken up by phagocytic cells and these *leishmania*-infected host cells produce proinflammatory cytokines that activate other immune cells and these infected host cells produce more cytokines and reactive nitrogen and oxygen species for efficient control of *leishmania* infection. Many experimental studies showed that resistance to infection with *leishmania* paraites is associated with the production of proinflammatory cytokines and activation of CD4^+^ Th1 response. On the other hand, vulnerability to this parasitic infection is correlated to production of T helper 2 cytokines that facilitate persistence of parasites and disease progression. In addition, some studies have also indicated that CD8^+^ T cells play a vital role in immune defense through cytokine production and their cytotoxic activity and excessive production of proinflammatory mediators promote amplified recruitment of cells. This could be correlated with excessive inflammatory reaction and ultimately resulted in tissue destruction and development of immunopathogenesis. Thus, there are contradictions regarding the role of immune responses in protection and immunopathogenesis of CL disease. Therefore, the aim of this paper was to review the role of host immune response in protection and its contribution to disease severity for CL infection. In order to obtain more meaningful data regarding the nature of immune response to *leishmania*, further in-depth studies focused on immune modulation should be conducted to develop better therapeutic strategies.

## 1. Introduction

Leishmaniasis is one of the most important vector-borne diseases caused by diverse intracellular protozoan parasites under the genus *Leishmania*. The disease spread to humans with infected female sand flies' bite that inoculates infective promastigotes into the mammalian host and displays various clinical manifestations, ranging in severity from less severe (self-healing cutaneous) to the most severe forms of (fatal viscera) leishmaniasis [1, 2]. Various epidemiological studies revealed around 20 *Leishmania* species which have the ability to cause leishmaniasis in individuals. The disease is distributed worldwide and common in 102 countries, and most of them are developing countries including Ethiopia. Globally, an estimated 12-15 million people are affected and roughly, more than 350 million population are at high risk [3, 4].

Cutaneous leishmaniasis (CL) is the most common form of the disease around the globe. In general, CL is caused by various *Leishmania* species such as *L. major*, *L. tropica*, *L. mexicana*, *L. braziliensis*, *L. guyanensis*, and *L. amazonensis.* In our country, CL is triggered through infection with *Leishmania aethiopica* (*L. aethiopica*) [5, 6]. *Leishmania aethiopica* is endemic in East Africa, mainly in Ethiopia, and causes three various forms of CL (localized, diffused, and mucocutaneous) as indicated in (Table 1). LCL is a localized form of CL which is particularly manifested with a protective immune response restricting the parasite to the inoculation site, and DCL is a nonulcerative disfiguring, disseminated lesion and resembles leprosy described by poor cellular immune response allowing uncontrolled spread of the parasite leishmania. On the other hand, MCL is the progressive destructive ulceration of the mucosa and metastasis [7, 8]. The various clinical manifestations appeared to be determined predominantly by the host immune response and the parasite species [9–12].

Upon entry of *leishmania* promastigotes into the body of the host, the inoculated promastigotes are engulfed by innate immune cells, such as macrophages, neutrophils, and dendritic cells (DCs) [13, 14]. The promastigotes are able to persist in these phagocytic cells since they do have conceived machineries to evade the hosts' immune response endeavors at controlling the parasite progress and disease establishment [15] and they transform into amastigote forms of the parasite and proliferate in macrophages and spread to other macrophages depending on various parasite and host factors. The life cycle is completed while the sand fly feeds on a host, and the amastigotes enter the midgut of the sand fly [16–18].

Immunity to *leishmaniasis* is mainly initiated by way of innate immune cells followed by cell-mediated immune response. Innate cells are actively involved to respond against *Leishmania* infection, and they are well-appointed with numerous germline-encoded pattern recognition receptors (PRRs) [19], and the participation of adaptive immune response plays a central role to provide immune protection to *leishmania* parasite infection [20]. Coordinated interactions among the mechanisms of cell-mediated immune cells and initiation of targeted T cell populations during infection facilitate cytokine production, and this will activate the infected immune cells [21].

The host immune response in CL is also implicated both in protection and immunopathology, which means it may accelerate cure [22, 23]. Intensified T cell response and its amplified cytokine production despite low number of parasites facilitated mucosal CL (MCL) pathogenesis and resulted in development of mucosal lesions [24, 25]. Therefore, there are several contradictions that continue about the role of immune responses in immunoprotection and immunopathology of CL infection. Therefore, the objective of this seminar paper is to review the role of host immune response in protection and its contribution to disease severity during CL infection.

## 2. Immune Response to *Leishmania* Infection

### 2.1. Innate Immune Response in CL Infection

The host innate immune response to CL is mediated by natural killer (NK) cells, macrophages, DCs, neutrophils, cytokines, and chemokines as well as the complement proteins [11]. In particular, macrophages, DCs, and neutrophils are the primary host immune cells that are recruited to the site of infection early after infection and could become infected by the parasites. These innate cell receptors recognize surface molecules present on the parasite surface, such as lipophosphoglycan (LPG), glycoprotein-63 (gp63), and glycosylphosphatidylinositol (GPI) and induce production of proinflammatory cytokines as well as costimulatory molecules [26]. This will in turn further enhance *leishmania*-infected host cells to produce reactive oxygen species (ROS) and nitric oxide (NO) which are more efficient mechanisms responsible for controlling l*eishmania* infection, and they play a critical role in shaping the immune response to *leishmania* parasite infection [27, 28].

#### 2.1.1. The Involvement of Macrophages and Dendritic Cells in CL Infection

**Figure 1:**
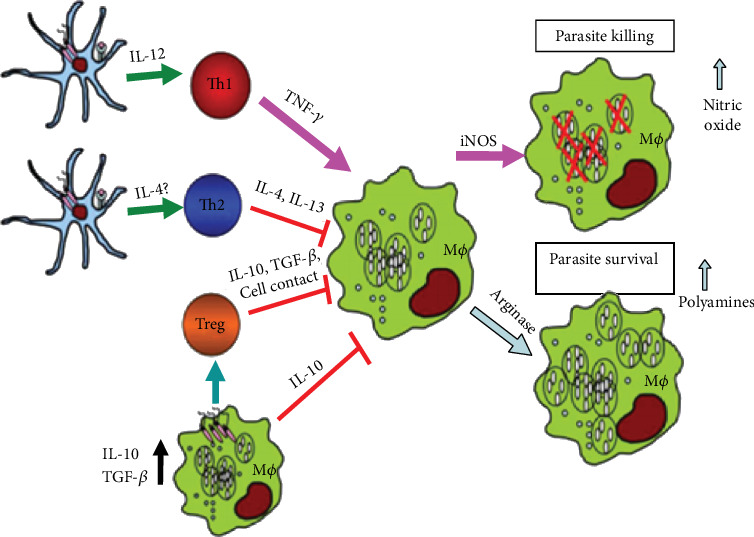
Dendritic cells and macrophages regulate the outcome of *Leishmania* infection (source: Liu and Uzonna, 2012).

Macrophages and DCs are among the primary host cells engaged to the site of infection and come to be infected by *leishmania* parasite [29]. Through a toll-like receptor-9- (TLR-9-) dependent pathway, infected macrophages and DCs play an important role in the production of IL-12 leading to activation of NK cells to produce interferon gamma (IFN-*γ*) and tumor necrosis factor alfa (TNF-*α*). These NK cell-derived cytokines play a more prominent role in host immune response by activating infected macrophages and DCs (Figure 1) [29]. In addition, IFN-*γ* and TNF-*α* could be produced from activated CD4^+^ Th1 cells and these cytokines are involved in activating *leishmania*-infected host cells as well. These activated parasite-infected cells are capable of producing reactive oxygen intermediates (ROI) or reactive nitrogen intermediates (RNI), which are the primary mechanisms of macrophages and DCs for efficient killing of intracellular *leishmania* parasites. Furthermore, activated macrophages and DCs produce enhanced cytokines (IFN-*γ*, TNF-*α*, and IL-12) that will further activate many immune cells [30]. Nitric oxide (NO) is a powerful cytotoxic molecule that plays a major role in killing many intracellular pathogens, including *Leishmania* parasites. In CL infection, macrophages, therefore, play a triple role since they are host cells and antigen-presenting cells (APC) that activate specific T cells and effector cells whose leishmanicidal efficiency can be determined by the existence of activating cytokines such as IFN-*γ* and TNF-*α* as well as they also serve as a major site of parasite replication [31].

#### 2.1.2. The Role of NK Cells in CL Infection

Natural killer (NK) cells are important innate immune cells which are defined classically as CD3^−^CD16/56^+^ cells [32]. Their role is realized via cytotoxic activity and production of early cytokine and chemokines before the adaptive immunity is induced that could assist in directing the immune response towards Th1, which is crucial for effective control of the parasites [26]. Several experimental investigations have indicated that early activation of NK cells is mediated by cytokines mainly IL-2 (secreted from Ag-specific CD4^+^ T cells), IL-12, TNF-*α* and IFN-*γ* produced from infected macrophages and DCs as well as chemokines [29, 33].

Once NK cells are activated, they do have the capability to directly lyse *Leishmania-*infected macrophages and DCs that express an altered cell surface phenotype through upregulation of ligands for NK cell-activating receptors (e.g., NKG2D) (either by direct killing of extracellular microbes or by inducing the death of intracellular pathogens via the transfer of perforin or granulysin) [34, 35]. In addition to their cytotoxic potential, NK cells also function as producers of cytokines such as IFN-*γ* and TNF-*α*, which contribute to activate macrophages and DCs to kill the intracellular parasites. These cytokines also play a critical role in directing the adaptive immune cells towards CD4^+^ Th1 response [36, 37].

In human leishmaniasis, NK cells were detected in lesions of patients with LCL, diffuse CL (DCL), and MCL cases [38]. Twenty-four hours after infection with *L. major* infections of self-healing C57BL/6 mice, NK cells' cytotoxic activity and IFN-*γ* production became readily detectable in the draining lymph node [39]. In addition, NK cells purified from unexposed human peripheral blood mononuclear cells (PBMCs) were cocultured with *leishmania* antigen and these cells proliferated and secreted IFN-*γ* in response to antigen [40]. From this experimental demonstration, one can conclude that NK cells proliferating in response to *Leishmania* antigen stimulation are involved in protection in healing of CL infection. Moreover, it has also showed that the depletion of NK cells within the first 7 days of *L. major*-infected mice leads to significant reduction in IFN-*γ* production and higher parasite burden indicating an important role of NK cells during the early immune response to *Leishmania* infection [41].

#### 2.1.3. The Role of Neutrophils in CL Infection

Early after host infection with *Leishmania*, neutrophils are the first cells which are rapidly and massively recruited to the site of *Leishmania* infection and release several factors including neutrophil extracellular traps (NETs), cytokines, and chemokines [42–44]. In murine models, the rapid recruitment as well as prolonged infiltration of neutrophils at the site of inoculation of promastigotes by sandflies within the first hours of infection with *L. major* was detected [42].

**Figure 2:**
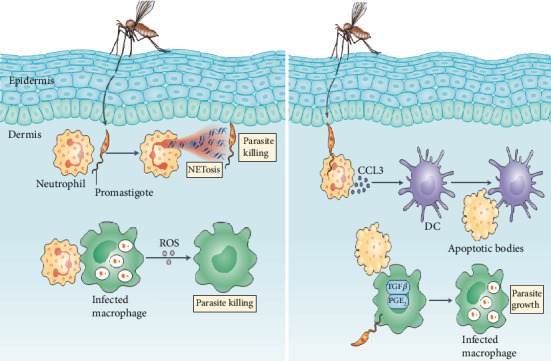
The role of neutrophils in leishmania infection control and persistence of infection (adopted from: http://www.nature.com/nri; Phillip Scott and Fernanda O. Novais, 2016).

Neutrophils also produce CC-chemokine ligand 3 (CCL-3) early after infection with *Leishmania* parasites, and this chemokine promotes the recruitment of macrophages and DCs to the site of infection (Figure 2) that participate in the phagocytosis of apoptotic infected neutrophils. Conversely, uptake of these apoptotic neutrophils by macrophages and DCs could limit the activation of those cells, which results in an attenuation of *Leishmania* antigen presentation, expression of surface activation markers, and inefficient activation of Th1 cells and CD8^+^ T cells [45]. Infected neutrophils are stimulated by the parasite to produce high levels of monocyte-attracting chemokine (MIP-1*β*), and eventually, those *leishmania*-infected neutrophils become apoptotic, and uptake of these dying neutrophils by macrophages results in the secretion of anti-inflammatory cytokines such as IL-10 and TGF-*β*. This will reduce production of proinflammatory cytokines such as IFN-*γ* and TNF-*α*. This condition creates an anti-inflammatory environment which promotes parasite survival in macrophages [46].

However, other experimental studies investigated a negative role of neutrophils in murine leishmaniasis, producing more severe disease associated with a Th2 response, or even serving as a carrier for *Leishmania* entry into macrophages [47]. It has been shown that absence of neutrophils had a protective effect associated with a reduced Th2 response and partial resolution of lesions in susceptible BALB/c mice infected with *L. major*. Furthermore, researchers showed that neutrophils may serve as vectors for the entry of *Leishmania* into macrophages [46]. It has been observed that macrophages phagocytose infected neutrophils in vitro which appear to deliver viable organisms to the macrophages [20]. Thus, *Leishmania* employs neutrophils as “Trojan Horses” to infect macrophages and DCs and establish the initial phase of cutaneous infection.

### 2.2. Humoral Immune Response to CL Infection

Human being infected with *Leishmania* species is demonstrated by the manifestation of anti-*leishmania* antibodies, which are produced at low level in CL and at a very high level in VL, which play no role in protection. However, analysis of *Leishmania* antigen-specific immunoglobulin isotypes in CL and VL patient sera revealed elevated levels of IgG and IgG subclasses compared to controls [48, 49] and it seems that the level of antibody response appears to reflect the parasite density and the intensity of the infection [50]. Generally, a high antibody level is a marker of progressive disease in visceral leishmaniasis [51], whereas the role of antibody titers in resolution of CL and protective immunity is largely unknown [52].

### 2.3. Cell-Mediated Immune Response in CL Infection

Since *Leishmania* parasites are intracellular pathogens, cell-mediated immune response is predominately crucial for efficient restriction of the infection with these parasites and hence, T cells are indispensable for resistance development [53]. Experimental investigations have shown that mice that lack T cell were highly vulnerable to various *Leishmania* parasite species infection, and adoptive transfer of T cells restores resistance in these infected mice. In this case, both CD4^+^ and CD8^+^ T cells were very critical for optimal primary immune protection to *L. major* though their relative contribution may depend on the host immune status and parasite strains/species [54]. The activation of targeted T cell populations for appropriate cytokine production is very important for coordinated interactions among cells [21].

#### 2.3.1. The Role of CD4^+^ TH Cells in CL Infection

Human CL have displayed that CD4 T cells are critically important for control of *Leishmania* infections because they serve as the core home of cytokines like IFN-*γ* that triggers various cells [55]. Phenotypic analysis of lymphocyte activation status in CL infection revealed significant increase of CD4^+^ T cells more prominent in acute phase of the infection [56]. In addition, particular phenotypic characterization of CD4^+^*Leishmania-*reactive T cells from *L. braziliensis* stimulated cells in patients with CL infection and revealed higher proportions of CD4^+^ as compared with CD8^+^ antigen*-*reactive T cells during active CL [57]. The outcome of infection is mainly dependent on the activation of one of the two subsets of CD4^+^ T cells, Th1 and Th2 cytokines, that activate macrophages and DCs [58].

**Figure 3:**
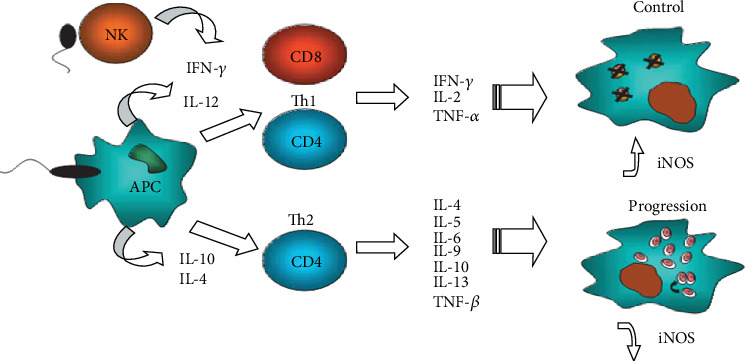
CD4^+^ Th1vTh2 cell response towards *Leishmania* infection (source: https://www.google.com; J. H. Ruiz & I. Becker (2007): CD8 cytotoxic T cells in cutaneous leishmaniasis).

In an experimental infection of mice which were deficient in CD4^+^ Th1 cytokines, IL-12, IFN-*γ*, TNF-*α*, or inducible NOS (iNOS) revealed failure to control parasite replication [59]. The balance between Th1 and Th2 cell responses is critical in determining disease severity [60–63]. In the mouse model infected with *L. major*, it has been established that a Th1 response leads to cure of the disease, whereas a Th2 response leads to disease progression [10]. Furthermore, in human CL, CD4^+^ Th1 immune response with the production of IFN-*γ*, TNF-*α*, and IL-12, as depicted in (Figure 3), has been correlated with infection control via macrophage activation and parasite destruction [11, 64]. Similarly, mouse strains that are typically susceptible to infection were treated with IL-12, and later on, they developed a Th1 response and become resistant to *leishmania* infection [37].

In *leishmania* infections, Th17 responses can also be detrimental to the host through acting on many cells and these activated cells release many mediators that could mediate tissue damage [65]. Enhanced IL-17 production in CL patients infected with *L. braziliensis* was observed through directly correlating the magnitude of cellular infiltrate with elevated levels of IL-17 production [66]. Furthermore, significant increase in IL-17 production was detected in culture supernatants of PBMCs from cutaneous and mucosal leishmaniasis patients after stimulation with Soluble *Leishmania* Antigen (SLA) in signaling deficient (IL-10SD) mice. However, when IL-10 was included in the culture, there was a significant decrease in IL-17 production levels by culture supernatant cells from both types of patients, but high levels of IL-17 production by PBMCs from mucosal patients compared with cutaneous *Leishmania*-infected patients [67, 68].

#### 2.3.2. The Role of CD8 T Cells in CL Infection

CD8^+^ T cells play a major role in immune protection for a widespread intracellular pathogens, including viruses, bacteria, and protozoan parasites. However, in case of CL infection, their role could be associated with both protective immune response and mediation of immunopathogenesis [69]. Some studies in experimental models and human infection with *leishmania* parasites have demonstrated that CD8^+^ T cells play a vital role in immune protection through cytokine production (IFN-*γ*, TNF-*α*) as well as their cytotoxic activity. There are controversies as regards the route of activation of CD8^+^ T cells following recognition of *leishmania* antigen. However, several studies support that external or secreted *Leishmania* antigens are able to reach the macrophage cytosol and degraded by proteasome enzymes and transported into the endoplasmic reticulum, where the peptides are bound to MHC class I molecules. Then, this MHC-I bound to the leishmanial peptides will be transported through the Golgi apparatus to the cell surface to be recognized by CD8^+^ T cells [70, 71].

The production of cytokines by CD8^+^ T cells is crucial to polarize CD4 T cells towards a protective Th1 immune response [72, 73]. However, the cytokine production by CD8^+^ T cells is lower in frequency and intensity as compared to CD4^+^ T cell subpopulations [74], whereas the cytolytic activity of CD8^+^ T cells is mediated by expressing cytolytic molecules such as perforin and granzyme, thereby initiating apoptosis of *leishmania* parasites through activation of caspase enzymes [75]. The role of CD8 T cells in *L. mexicana*-caused LCL and DCL in patients has been investigated. In this study, CD8^+^ T cells have exhibited cytotoxic activity and cytokine production (IFN-*γ*) in the case of LCL patients, but in the case of DCL patients, these cells revealed reduced cytotoxicity and cytokine production capacity, which could be associated with chronic state of CD8 functional exhaustion and facilitate disease progression [76].

Furthermore, a number of CD8 T cells have been detected in the lesions as well as in peripheral blood of CL patients. In the same way, CL patients infected with *L. major* and *L. mexicana* have also demonstrated the same situation during healing process [57, 77]. Another study indicated that lesions of patients with LCL infected with *L. braziliensis* exhibited a large number of CD8 T lymphocytes in apoptosis; conversely, patients undergoing a spontaneous cure presented very few apoptotic CD8 T cells [75, 78]. The overall inference of these data shows that CD8^+^ T cells participate in active disease and could be correlated with cure. In addition to cytokine production, CD8 T cells participate in the control of *Leishmania* infection through cytotoxic mechanisms including perforin and granzymes [75].

In patients with LCL and MCL infected with *L. braziliensis*, it has been investigated that CD8^+^ T cells have been associated with tissue damage. These primed CD8 T cells have the capacity to lyse autologous infected macrophages [24, 79, 80]. Some studies showed that many CD8^+^ T cells were found in the lesions of *L. braziliensis*-infected patients and these cells expressed cytolytic markers, such as CD107 and granzyme B^+^ and the frequency of granzyme B^+^ expression in CL lesions were positively correlated with lesion size. In addition, biopsies from these patients have also exhibited a more intense process of necrosis; a higher percentage of granzyme B^+^ cells were observed. Thus, they conclude that CD8^+^ T cells participate in the healing process as well as CD8^+^ granzyme B^+^ T cells mediate tissue injury [69].

#### 2.3.3. The Role of Regulatory T Cells in CL Infection

Regulatory T cells (T regs) are a specialized CD4^+^CD25^+^Foxp3^+^ T cell subpopulations that suppress the activation and effector function of various immune cells and thereby maintain homeostasis of the immune system and tolerance to self-antigens [81]. These cells develop in the thymus and are defined by expression of a transcriptional factor FoxP3^+^ which is required for T reg development and appears to control a genetic program specifying this cell fate and plays a significant disease-controlling role [82, 83]. In case of CL infection, T regs are crucial for suppression of detrimental immune responses especially to self-antigens; however, they may also lead to the suppression of beneficial immune responses of the host. These cells are responsible for producing regulatory cytokines such as TGF-*β* and IL-10 which act back on macrophages and DCs to reduce the release of inflammatory mediators, forming a negative feedback loop and the balance of pro- and anti-inflammatory cytokines and control immunopathology and tissue destruction at the site of *leishmania* infection site [69].

An experimental study showed that accumulation of T regs during *L. major* infection in both human and mouse models showed that these cells suppress parasite elimination by CD4^+^CD25^−^ effector T cells so that they mediate disease chronicity and also their depletion leads to parasite clearance by other CD4^+^ T cells [59, 84]. Similarly, another investigation showed that depletion of CD25^+^ (high) cells in this mouse strain augmented the production of IFN-*γ* by CD4+ T cells in the lesions that could have resulted in reduction of the parasites [85].

An experimental study was carried out from skin biopsy samples to evaluate the role of natural regulatory T cells (nTregs) in early and late cutaneous lesions of human infected with *L. major*, and it was investigated that the mean expressions of Foxp3 mRNA and also protein staining of natural T reg markers in lesions of patients with *L. major* infection were significantly increased in chronic lesions than early lesions [86]. Similarly, T regs were also detected from lesions of *L. major*-infected C57BL/6 mice and they responded to *L. major* antigen and accumulate rapidly at the site of infection and suppress other CD4+ T cell activity, which favors parasite persistence [87, 88].

#### 2.3.4. Cytokine Profile in CL Infection

Cytokines are chemical messengers that convey information between and within the immune system through specific cell surface receptor molecules. They play a key role in modulation of immune response against *leishmania* parasite infection, presenting local and systemic effects, and determine the resistance or susceptibility nature of the disease [89, 90]. Their production is transient, and they contribute diverse roles in various cells including activation, proliferation, cell differentiation, cell recruitment, and release of effector molecules and do have multiple effects on various cells. These cytokines could be either proinflammatory cytokines or anti-inflammatory cytokines [21, 91].

**Figure 4:**
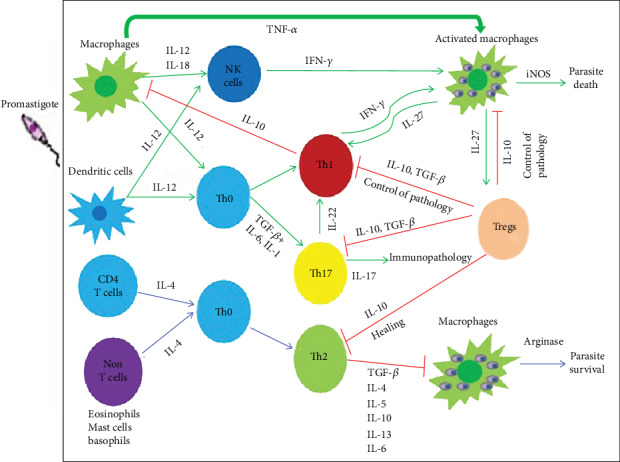
Cytokine profile in cutaneous leishmaniasis infection (Source: Pathogens and Global Health; Maspi et al.).

Proinflammatory cytokines are produced primarily for amplifying the immune response to *Leishmania* infection. The major proinflammatory cytokines include TNF-*α*, IFN-*γ*, IL-1, IL-2, IL-8, IL-12, IL-15, IL-18, and IL-17, whereas anti-inflammatory cytokines are immunoregulatory molecules that counteract the effects of proinflammatory cytokines to limit the inflammation that is triggered by excessive production of proinflammatory cytokines as indicated in (Figure 4). These major anti-inflammatory cytokines include IL-5, IL-6, IL-4, IL-10, IL-13, and TGF-*β* [92]. Experimentally, it has been investigated that IFN-*γ*-deficient C57BL/6 mice infected with *L. amazonensis* showed larger lesions, increased parasite burden, and development of Th2 type immune responses associated with IL-4 elevations as compared with wild-type mice [93]. Therefore, cytokines determine the resistance or susceptibility nature of the disease and the balance in the production of proinflammatory and regulatory cytokines determines the profile of immune response and influence on disease severity [94]. Thus, it seems that the outcome of infection could depend on whether the host mounts primarily Th-1 inflammatory cytokines (especially IFN-*γ*, TNF-*α*, and IL-12) which are crucial factors in the initiation of immune protection or Th-2 inflammatory cytokines facilitate the persistence of parasites through downregulating Th1 immune response to *leishmania* infection [21, 95]. Administration of cytokines is a possible approach for modifying biological effects associated with immune diseases. Hence, the cytokines may address as potential therapeutics in the future.

## 3. Immunopathogenesis of Cutaneous Leishmaniasis

The immune response processes require tight regulation to avoid uncontrolled amplification of the immune response, which may lead to immunopathology. *Leishmania* parasites have devised various mechanisms to circumvent the hosts' endeavors at restricting parasite growth and disease establishment [20]. In contrast to their protective roles, excessive production of proinflammatory cytokines and chemokines promotes the expression of adhesion molecules, leading to the amplified recruitment of cells from the blood. This ultimately will enhance inflammation that could result in tissue destruction and disease severity [96]. In the case of impaired T reg function during CL infection, increased production of proinflammatory cytokines could be associated with excessive inflammatory reaction and eventually resulted in tissue destruction and development of the lesions following *Leishmania* infection. In CL and MCL patients infected with *L. braziliensis*, an experimental investigation has revealed that production of these cytokines (IFN-*γ* and TNF-*α*) and decrease production of regulatory cytokines (IL-10 or the IL-10 receptor) lead to an exaggerated inflammatory response that was responsible for development of immunopathogenesis [25, 97, 98].

Another mechanism for the development of immunopathology of CL infection is due to the active involvement of IL-17, which is a strong proinflammatory cytokine that is secreted primarily by activated CD4^+^ Th17 cells and neutrophils and has been implicated in several important inflammatory human diseases including human leishmaniasis infection [44, 99]. It intensifies an ongoing tissue inflammation through acting on a broad range of cells to induce the expression of cytokines (IL-6, IL-8, TNF-*α*, IL-1*β*, granulocyte colony-stimulating factor (G-CSF), and chemokines (CXCL-1, CXCL-10) [35]. Enhanced IL-17 production in patients infected with *L. braziliensis* was observed through directly correlating the magnitude of cellular infiltrate with elevated levels of IL-17 production [66]. A similar study has also showed elevated IL-17 levels in PBMC culture supernatants in active *L. braziliensis* human CL infections [100].

Significant increase in IL-17 production was detected in culture supernatants of PBMCs from LCL and MCL patients infected with *L. major* in IL-10-deficient mice and contributed to the pathogenesis of infection. However, when IL-10 was included in the culture, there was a significant decrease in IL-17 production levels from both types of patients and the pathology was reduced and this study concludes that increased production of IL-17 mediates extensive immunopathology if not regulated by IL-10 [67]. CD8 T cells have also been implicated in the chronicity of *Leishmania* infections by exacerbating the tissue lesions possibly through expressing granzyme B and Perforin. Gene expression profiling of skin lesions from CL patients infected with *L. braziliensis*, using both human samples and mouse models, showed that activation of CD8^+^T cell cytolytic responses was detrimental to the host through increased immunopathology, which was associated with enhanced recruitment of neutrophils to the site of infection. In the case of MCL patients, these cells also enhanced the development of metastatic lesions distant from infection sites due to a destructive inflammatory response [101].

The other important risk factor for the development of immunopathology due to this parasite infection is Leishmania RNA virus (LRV), which is a key virulence factor associated with the development of mucocutaneous leishmaniasis [102]. These viruses mediated disease exacerbation that relies on toll-like receptor 3 (TLR3) activation, but downstream mechanisms remain largely unexplored. However, human and mouse model data demonstrated that patients infected with LRV+ parasites displayed lower levels of IL-1*β* and active Casp1 as compared with patients infected with LRV− parasites. Although TLR3 has a minor role in detecting and responding to Leishmania spp. compared with TLR9, upon *L. guyanensis* LRV+ infection, TLR3 senses dsRNA from LRV in the endosomal compartment, triggering a robust inflammatory response, with the production of TNF-*α* and type I IFN, exacerbating disease in mice [102, 103]. Various experimental study results indicated that inflammasome activation is directly correlated with disease severity, and the presence of the LRV influences inflammasome activation and disease development. These data prompted us to further investigate the mechanisms underlying these processes [104].

In *Leishmania guyanensis*, the nucleic acid of *Leishmania* RNA virus (LRV1) acts as a potent innate immunogen, eliciting a hyperinflammatory immune response through toll-like receptor 3 (TLR3). The resultant inflammatory cascade has been shown to increase disease severity, parasite persistence, and perhaps even resistance to antileishmanial drugs [105]. In conclusion, several study findings revealed the mechanisms triggered by LRV that contribute to the development of the debilitating mucocutaneous form of leishmaniasis. Moreover, LRV promotes disease severity and parasite survival and promotes degradation of NLRP3 and ASC via autophagy; TLR3 mediates LRV-induced inhibition of inflammasome activation.

Extracellular vesicles (EVs) are a heterogeneous group of particles that are released by cells and play a pivotal role in intercellular communication [105]. Proteins, glycoconjugates, RNA, DNA, lipids, and metabolites are present in EVs and can be easily transferred from one cell to another. Those extracellular vesicles (EVs) released by *Leishmania* can contribute to the establishment of infection and host immunomodulation [106, 107]. Some studies have reported that extracellular vesicles derived from *Leishmania* can contribute to immune evasion, pathogen survival, and disease progression [108]. Pretreatment of C57BL/6 mice with exosomes from *L. donovani* increased the parasite load and generated an immune response with suppressor characteristics [109]. Similarly, BALB/c mice showed an exacerbated disease progression and Th2 polarization after inoculation with exosomes prior to infection with *L. major* [108]. Mice coinjected with *L. major* and exosomes derived from parasites cultured *in vitro* or derived from sand fly had a significant exacerbation of the lesions and higher mRNA expression of IL-2, IL-4, IL-17A, IL-23, IL-10, and IFN-g in draining lymph nodes [110]. Therefore, as demonstrated by other *Leishmania* species and parasites, the extracellular vesicles released by *L. amazonensis* modulate the immune response to favor the parasite growth and disease development [111]. Moreover, in *Leishmania guyanensis*, the nucleic acid of *Leishmania* RNA virus (LRV1) acts as a potent innate immunogen, eliciting a hyperinflammatory immune response through toll-like receptor 3 (TLR3). The resultant inflammatory cascade has been shown to increase disease severity, parasite persistence, and perhaps even resistance to antileishmanial drugs [112].

## 4. Conclusions

The most important determinants for a broad spectrum of clinical manifestations of CL infection are due to variation in *Leishmania* species and the host immune status towards parasites' antigens. The immune response to *leishmania* infection is primarily initiated by innate immune cells followed by cell-mediated immune response. Both innate and cell-mediated immune response are predominately crucial for efficient control of *leishmania* infection, and hence, T cells are vital for resistance development. Beside its immunoprotection to *leishmania* infection, their amplified production may concurrently lead to immunopathogenesis. Administration of cytokines could be a possible approach for modifying biological effects associated with immune diseases, and they could be potential therapeutics in the future. In order to obtain more meaningful data regarding the nature of immune response responsible to cure or susceptibility to CL infections, further in-depth studies from mouse models to human infection focused on the immune modulation should be conducted to develop better therapeutic options and vaccine strategies to this most important neglected tropical disease.

## Figures and Tables

**Table 1:** Leishmania species, the disease form they can cause, their geographical distribution, and vectors are summarized.

Leishmania species	Clinical form in humans	Geographical distribution	Vectors
*Leishmania aethiopica* ^∗^	Localized cutaneous leishmaniasis Diffuse cutaneous leishmaniasis	Ethiopia, Kenya	*Phlebotomus longipes* *P. pedifer*
*L. major* ^∗^	Localized cutaneous leishmaniasis	North Africa, Middle East, Sub-Saharan Africa, and Sahel belt, Sudan, Pakistan	*P. papatasi* *P. duboscqi*
L. mexicana^∗^	Localized cutaneous leishmaniasis	Central America	*Lutzomyia olmeca*
*L. amazonensis* ^∗^	Localized cutaneous leishmaniasis	South America, north of the Amazon	*L. flaviscutellata*
*L. braziliensis* ^∗^	Localized cutaneous leishmaniasis Mucocutaneous leishmaniasis	South America, Central America, and Mexico	*Psychodopygus Lutzomyia* spp.
*L. peruviana* ^∗^	Localized cutaneous leishmaniasis	West Andes of Peru, Argentine highlands	*L. verrucarun* *L. pvmenis*
*L. infantum* ^@^	Visceral leishmaniasis Localized cutaneous leishmaniasis	Middle East and Central Asia to Pakistan, China, Central and South America, Southern Europe, northwest Africa	*P. pcrniciosufi* *P. arias*
*L. donovani* ^@^	Visceral leishmaniasis	Ethiopia, Sudan, Kenya, India, China, Bangladesh	*Phlebotomus argentipes* *P. orientalis*

^@^Old World species. ^∗^New World species.
